# Editorial: Proactive work design in unstructured work: New challenges and opportunities

**DOI:** 10.3389/fpsyg.2023.1087740

**Published:** 2023-01-24

**Authors:** Arianna Costantini, Hai-Jiang Wang, Keri A. Pekaar, Piet van Gool

**Affiliations:** ^1^Department of Psychology and Cognitive Science, University of Trento, Trento, Italy; ^2^School of Management, Huazhong University of Science and Technology, Wuhan, China; ^3^Department of Human Resource Studies, Tilburg University, Tilburg, Netherlands; ^4^Department of Industrial Engineering and Innovation Sciences, Eindhoven University of Technology, Eindhoven, Netherlands

**Keywords:** hybrid work, job crafting, job design, new ways of working, proactive work behaviors, remote work

## Introduction

The pandemic lit up a revolution in the world of work that arrived almost overnight but whose impact is still alive and seems to be here to stay (Becker et al., 2022). The significant changes in the experience of work spanned from its more tangible aspects, with the abrupt transition to remote and then hybrid work, to its relational components and a profound redefinition of the meanings of work. Within this new context, employees have to find their own ways to deal with remote collaboration, cope with insecurity, and shape new meanings of the boundaries between work and personal domains (Gino and Cable, 2020).

In these times of change, in this Research Topic we aimed to deepen our understanding of the ways in which organizations and employees navigate this unprecedented situation and use agentic change to create the conditions that work best for them, reinventing work and its configurations. We are excited to introduce nine articles presenting research from all over the world that unpack a complex net of processes unfolding at different levels of analysis and across different contexts to create the future of work.

## Articles

### Organizational policies and work characteristics as influences

Widening the scope of autonomy in carrying out work, de Bloom et al. present a conceptual review on unlimited paid time off policies (UPTO) through which employees have the opportunity to take time off from work whenever desired while receiving their full wage. In their review, they present and discuss the psychological and social mechanisms linking UPTO to potential beneficial outcomes and unintended detrimental social consequences that may result from unbound autonomy.

Zooming in on the context of telework, Liu et al. focus on how to ensure that employees with distributed work arrangements still feel attached to their organizations. Their three-wave study attests the key role of feedback quality for teleworkers' experience of organizational support—a link that is particularly important for conscientious teleworkers—which then promotes a higher sense of belongingness toward the organization.

Bai et al. acknowledge the increasing complexity that characterizes contemporary jobs and investigate how complexity can represent a trigger of motivational states and energy levels. Their three-wave study shows that jobs that are mentally demanding and challenging push employees to craft their tasks and relationships in approach-oriented ways due to their high motivational potential. They also show that such high complexity can result in withdrawal-oriented behaviors when employees' energetic resources are depleted due to the high job complexity.

Further enriching our understanding of how job complexity is linked to proactive employee behaviors, Schmitt examines the role of employees' awareness and processing of sensory information and their reactivity to internal and external stimuli as boundary conditions influencing proactive work behaviors. Her results show that employees displaying high awareness and openness to the positive aspects of one's surroundings engage in proactive work behaviors more often and are better able to translate higher job complexity into opportunities to be more proactive at work than employees who are less aware of their surroundings.

### Employees shaping their experiences through personal proactivity

Focusing on proactive job redesign to promote engaging and significant work during remote work, Costantini and Weintraub present a weekly diary study on the dynamics of different strategies that employees use to shape the work conditions that fit them best. They show that employees who proactively build connections with others because they can self-regulate their work through self-goal setting reported higher significance in their work tasks. Moreover, in weeks when employees proactively expanded their social interactions due to the positive effects of higher self-goal setting, they reported higher weekly work engagement.

Li et al. further investigate how proactive strategies focused on building harmonious relationships at work can be linked to different work outcomes. Using two-wave data, they show that such proactive efforts may slow down work processes and impair employees' energy levels but also that crafting relational aspects can benefit a sense of personal connection—guanxi—at the workplace, which promotes higher work engagement and limits emotional exhaustion.

Providing insights on the timely topic of the great resignation, Xin et al. present a two-wave study investigating the role of employees' work proactivity in influencing turnover intentions and how perceived organizational instrumentality and inclusive leadership may be involved in such a relation. Their results show that employees who craft their jobs perceive the organization as a context providing them with opportunities to achieve their personal goals, which leads to lower turnover intentions. Importantly, this relation turned out to be weaker in the context of higher inclusive leadership. However, they also show that job crafting alone can lead to higher turnover intentions when organizational instrumentality is not accounted for, highlighting the importance of employees seeing their organization as a context to achieve their personal goals.

Expanding our understanding of the mechanisms underlying proactivity in the face of great uncertainty, Koen and van Bezouw present a three-wave study showing that feelings of job insecurity can prompt future focus among participants reporting high-income adequacy. Moreover, such a focus triggers greater engagement in proactive career behaviors that are associated with lower expected likelihood of losing one's job.

Finally, Kerksieck et al. shift the focus to the proactive crafting of the balance of work-to-nonwork interfaces. In five studies with participants from five different countries, they introduce a new instrument to measure how employees craft an idiosyncratic balance of work and nonwork domains and show that these proactive efforts are positively linked with wellbeing indicators and perceptions of work-nonwork balance.

## Conclusions and practical implications

The studies included in this Research Topic provide timely insights into understanding the work transformations brought about by the pandemic and how employees and organizations cope with these changes. In doing so, the contributions provide a set of empirical examples supporting recent theoretical frameworks on how the constellation of job characteristics can be transformed during times of crisis and on the intertwined roles of different actors when it comes to managing crisis to support employee health and motivation (Demerouti and Bakker, 2022). By analyzing the roles of both contextual and individual factors, the contributions presented here provide interesting insights into how effective crisis management depends on joint efforts from multiple stakeholders.

De Bloom et al. and Liu et al. highlight that in unstructured work, HR policies should be even more clear in expressing what they expect from their employees and how to support them. For example, when designing HR policies, organizations should be aware that UPTO policies have the potential to contribute to better work-nonwork balance but may also arouse feelings of uncertainty and guilt about the completion of work (de Bloom et al.). In a similar vein, when it comes to fostering a sense of attachment to the organization in the context of distributed work, high-quality feedback and perceived organizational support are essential keys for building teleworkers' sense of belongingness (Liu et al.).

Focusing on how jobs are designed when a clear structure is lacking, the studies from Koen and van Bezouw and Bai et al. show how times of crisis can increase the levels of job demands—job insecurity and job complexity, respectively—and the importance of (job) resources to effectively manage the additional effort. Highlighting the importance of a synergistic perspective to the management of uncertainty, Bai et al. and Schmitt show that while job complexity can represent a positive component of unstructured work leading to a gain spiral of proactive behaviors, especially for more sensitive employees, organizations should make sure to buffer the complementing psychological costs of proactivity with additional resources. The implication that follows is that the relevance of job resources becomes even more salient during such uncertain and complex times in preventing detrimental consequences and supporting employees' potential for proactivity (Demerouti and Bakker, 2022).

The studies included here also provide insights into how regulatory individual strategies function in the context of unstructured work settings. Self-rewards and relational crafting can be important strategies to build a sense of community when dealing with the challenges of remote working (Costantini and Weintraub). However, job and relational crafting in times of crisis may also have a double-edged nature: on the one hand, these strategies may improve work dynamics and foster perceptions of the organization as a context for personal development, on the other hand, they may also increase emotional exhaustion due to additional individual efforts and heighten turnover intentions because employees feel that they may be interesting for future employers (Li et al.; Xin et al.). Finally, Kerksieck et al. show that when employees use their personal initiative when confronted with managing work and nonwork duties in the face of blurred boundaries, this can lead to beneficial outcomes for their wellbeing and performance. Hence, it seems that, overall, proactive regulatory attempts can help individuals manage uncertainty by modifying the impact of demands and resources on relevant outcomes (Demerouti and Bakker, 2022). However, organizations should also be aware that such proactive efforts can imply individual costs that should be accounted for by the provision of additional resources.

We hope that this Research Topic stimulates future research exploring the processes and outcomes of how organizations and employees reinvent work to bounce forward during challenging times.

## Author contributions

AC drafted the first version of the editorial. All authors provided conceptual input and approved the final draft.
